# Access considerations for a COVID-19 vaccine for South Africa

**DOI:** 10.4102/safp.v62i1.5152

**Published:** 2020-10-29

**Authors:** Varsha Bangalee, Fatima Suleman

**Affiliations:** 1 Discipline of Pharmaceutical Sciences, School of Health Sciences, University of KwaZulu-Natal, Durban, South Africa

**Keywords:** COVID-19, pandemic, vaccines, pricing, transparency resolution

## Abstract

The novel coronavirus (COVID-19) pandemic is a defining point in human history, having far-reaching effects on all aspects of human life. In the race to find a vaccine, governments need to work collectively to ensure that any life-saving interventions are accessible and affordable to populations across the globe. This pandemic has created an opportunity for international cooperation in working on transparency issues both in terms of sharing manufacturing details to make devices for the diagnosis and treatment of COVID-19 and in terms of clinical trials for therapies that could prove to be effective against the disease.

## Race for a vaccine

The novel coronavirus (COVID-19) pandemic is a defining point in human history. Declared a pandemic by the World Health Organisation (WHO) on 11 March 2020, the virus has since affected the lives of more than 21 million people worldwide, having devastating effects across all sectors of human life.^1^ With limited treatments currently available, pharmaceutical companies and scientists around the world are working tirelessly to develop a safe and effective vaccine.

Several companies are competing to be the first to develop a COVID-19 vaccine, such as GlaxoSmithKline, Sanofi, Johnson & Johnson, Pfizer, Gilead, Regeneron, Moderna, Takeda, Inovio, CureVac and Novavax.^2^ However, regulatory approval is just the first step in determining safe patient access. There are several challenges related to the cost of development, sufficient supply and distribution and affordability parameters that need to be considered to effectively address this global pandemic.

Under normal circumstances, governments encourage new drug and vaccine development by offering companies exclusivity periods, that is, period to monopolise on sales. The COVID-19 pandemic, however, is no ordinary situation, and thus, granting exclusivity periods is no longer a ‘fait accompli’. Whilst many vaccine manufacturers have promised to sell vaccines on a not-for-profit basis or to keep prices low, past experience with medicine pricing has warned of price gouging and inflated costs.

Johnson & Johnson has indicated that should they develop an approved vaccine, they would make the first 1 billion doses available at a not-for-profit price; however, it is unclear how the vaccine will be priced thereafter. Price setting beyond this is likely to follow the dynamics of standard supply and competitive forces. Therefore, policymakers around the world need to pre-empt these situations by improving the transparency and regulation of vaccine pricing. Furthermore, with massive worldwide demand, a single supplier may not be able to ensure a continuous supply chain. Vaccines may therefore be supplied preferentially to those countries that can pay for vaccines upfront. Thus, the inability to afford vaccines by low- and middle-income countries with severely limited budgets introduces an ethical dilemma of withholding access to an essential medicine to countries that cannot afford it.

Several countries, for example, Germany, the United States, Canada and those within the European Union, have pledged more than a billion dollars of public funds towards the research and development of vaccines, diagnostics and other promising therapeutics for COVID-19.^3^ Taxpayer funds are essentially the backbone of research initiatives. Despite this, there is no indication that these countries have included binding affordability requirements as conditions to this funding.^4^ It is here that governmental interventions and improved regulation are required to ensure that public contributions result in a return on investments in the form of free or affordable medicines and vaccines, and not high profits for private companies. Too often, life-saving interventions are priced out of reach for people who need them the most and put financial strains on government budgets. Poor medicine and vaccine accessibility as a consequence of high medicine prices was a situation that only low- and middle-income countries were accustomed to. However, in recent years, high-income countries, such as the Netherlands and the United States of America, have begun to reel under the burden of increasing medicine, vaccine and health-related technology costs.

## South Africa’s dilemma

In the context of low- and middle-income countries, including South Africa, affordability of an approved COVID-19 vaccine is a serious concern and presents several questions. In countries with established universal health coverage systems, such as Australia, Canada and the United Kingdom, vaccinations are offered at no cost. The two-tiered healthcare system in South Africa requires additional consideration. Under this landscape, the public sector renders healthcare to 84% of the population, whilst 16% is covered by the private sector;^5^ hence, within these confines who will foot the bill for the vaccines? Within the framework of limited health budgets coupled with the quadruple burden of disease, government funding for COVID-19 interventions would entail an opportunity cost, namely possible termination of another existing treatment program. Alternatively, the South African government would need to secure external sources of funding for new COVID-19 treatments and vaccines; however, being a global pandemic, foreign aid during this time of crisis is likely to be scarce.

A different picture could emerge for the private sector. As pharmaceutical companies typically price medicines relative to their benefit and demand, the cost of the COVID-19 vaccine in the private sector could be exorbitantly high regardless of actual research and development costs. The perception is that this is generally done to recuperate profits lost to differential pricing between the two sectors in South Africa. The costs may unfortunately be transferred to the patients in the form of co-payments or higher health insurance premiums. To overcome this pandemic, South Africa has to look at its patent laws and overcome existing patent, trade secret, and other access barriers, and leverage on public-private partnerships (e.g. Global Alliance for Vaccines and Immunisation [GAVI]) for distribution solutions.

## An international solution

The vaccine market is predominantly dominated by an oligopolistic market structure. In a free market, vaccine prices are usually determined by their societal benefits as well as governments’ willingness to pay for them,^3^ leading to differential pricing systems, where wealthier nations generally pay more. This premise, however, creates uncertainty, particularly owing to the lack of transparency associated with public procurement of vaccines.^6^

The WHO launched a database (MI4A/) in 2014, which required publicly available data on vaccine price information. This was seen as an important first step towards vaccine price transparency.^7^ The database reports vaccine prices paid by various governments across the world. However, it does have limitations, that is, it only contains information on the region each country is located and which income category it belongs to (i.e. high, upper middle, lower middle or low), without disclosing the names of the countries themselves, thus preventing direct comparisons between countries, and once again preventing transparency.

Calls for the improvement of transparency gained much momentum in 2019. This led to the World Health Assembly (WHA), in 2019, endorsing the WHO’s strategy for access to medicines and vaccines, where enhancement of price transparency is seen as a key step.^8^ These include research and development costs, clinical trial results and costs, medicines patents, the real prices of medicines (viz. the production costs), actual investment funded by companies and actual investment funded by taxpayers and non-profit organisations. The COVID-19 pandemic will put this resolution to test. Transparency in terms of research and development and ultimately pricing will be tested.

The Costa Rica government has already started the process. On 23 March 2020, it called on the WHO to ‘undertake an effort to pool rights to technologies that are useful for the detection, prevention, control and treatment of the COVID-19 pandemic’.^9^ The WHO heeded this call and is working on a memorandum of understanding (MoU) on the intent to share rights for the 2020 WHA. In terms of this MOU, governments, companies and research organisations are to make available any patents, clinical trial data or any other intellectual property related to COVID-19. This information will then be accessible in a central database. The use of these data will be either free or subject to a ‘reasonable’ pricing fee.

Thus, should patent monopoly or exclusive rights be provided to drug companies who develop COVID-19 vaccines? In a pandemic situation, should it not be possible for multiple pharmaceutical companies to manufacture a COVID-19 vaccine and in this way help to keep the price low? In keeping with the transparency resolution, another mechanism would be to make transparent the contracts between the funding agencies and pharmaceutical companies. These contracts would need to include language about reasonable pricing and access to the vaccine. It is important to deal with the situation upfront, rather than after a company has set a price. It is thus important to heed the lessons from past experience with vaccine (and medicine) pricing sagas.

## Lessons to heed from past experience

A limited supply or sole proprietary rights to sell the vaccine introduces the threat of vaccine price gouging. This occurred in 2004 when the influenza vaccine produced by the Chiron Corporation was deemed to be unsafe in the United States and removed from circulation, meaning that France’s Sanofi Pasteur was initially the sole supplier of the flu vaccine in the United States.^3^ This shortage led to pharmacists purchasing the vaccine at inflated prices. Possible strategies to stave off price gouging include price-capping measures. To prevent a similar crisis, advocacy groups, policymakers and governments the world over need to prohibit the granting of exclusive licensing of a successful coronavirus vaccine to only one company. A monopoly could result in expensive vaccines that are inaccessible and a waste of public resources.^10^

Government funding plays a critical role in supporting the research and development of life-saving vaccines. An apt example of this is the Ebola outbreak that peaked between 2014 and 2015. Public sector researchers (from universities and research institutes) contributed heavily towards the development and manufacturing of early vaccine batches. Despite their contribution, a single private entity was given the exclusive rights to further develop the vaccine and take to market. This however allowed the companies to control the sale and price of the vaccine, with no assurance that the vaccine would be affordable to the public. The lesson for COVID-19 being that governments and researchers need to be more proactive in negotiating a fair price for patients and health systems upfront particularly as the likely requirements for annual vaccination of a large proportion of the global population present a lucrative market for pharmaceutical companies.

The recent approval by the Food and Drug Administration (FDA) of Remdesivir (leads to quicker recovery in COVID-19 patients with advanced disease) is another case to ponder. Remdesivir is a product of Gilead Sciences, Inc. This is the same company that manufactures the hepatitis C medicine called Sofosbuvir. The initial selling price of $84 000.00 per treatment course raised an outcry amongst activists globally, leading to voluntary licence agreements being signed. In the United States, however, Sofosbuvir costs $48 000.00 for a 24-week course, or about $1000.00 a pill, which is still too expensive for people. Gilead is looking at further voluntary licence agreements for Remdesivir. This time the company should be held accountable to ensure that affordability access is expanded to include all patients in all regions of the world, especially as the National Institutes of Health provided $6.5 billion in funding for basic research.^11^

## Conclusion

This pandemic is an opportunity for international cooperation on transparency issues both in terms of sharing manufacturing details to make devices for the diagnosis and treatment of COVID-19 and in terms of clinical trials for therapies that could prove to be effective. The final step would be to relook at pricing strategies and move towards the pricing of medicines for public good rather than investor gain. The exceptional circumstances of the COVID-19 pandemic call for an exceptional response and out-of-the-box thinking.
